# Survival trends in gastric cancer in Brazil: real-life data from a large cancer center

**DOI:** 10.3332/ecancer.2024.1706

**Published:** 2024-05-30

**Authors:** Angelo Borsarelli Carvalho Brito, Tiago Cordeiro Felismino, Diego Rodrigues Mendonca e Silva, Maria Paula Curado, Lais Corsino Durant, Rodrigo Gomes Taboada, Adriane Graicer Pelosof, Alessandro Landskron Diniz, Felipe Jose Fernandez Coimbra

**Affiliations:** 1 Department of Clinical Oncology, A. C. Camargo Cancer Center, Sao Paulo 01509-010, Brazil; 2 Hospital Cancer Registry, A.C. Camargo Cancer Center, São Paulo 01509-010, Brazil; 3 Department of Abdominal Surgery, A. C. Camargo Cancer Center, São Paulo 01509-010, Brazil; 4 Department of Endoscopy, A. C. Camargo Cancer Center, São Paulo 01509-010, Brazil

**Keywords:** gastric cancer, prognosis, epidemiology

## Abstract

**Background:**

Gastric cancer (GC) is the fourth leading cause of cancer deaths globally. There is a paucity of real-life data on GC in Brazil. Our study aimed to evaluate survival trends in gastric adenocarcinoma (GA) in a large cancer center in Brazil during 2000–2017.

**Methods:**

Based on our Hospital Cancer Registry Database, all individuals diagnosed with GA between 2000 and 2017, and treated at A.C. Camargo Cancer Center, were retrospectively included. The primary objectives were to describe the patient demographics, clinicopathological characteristics, treatment modalities and survival trends during four separate periods of diagnosis (2000–2004; 2005–2009; 2010–2014 and 2015–2017). *χ*2 test was performed between two specified periods (2000–2004 and 2015–2017) to compare categorical variables. Overall survival (OS) curves were stratified by four separate periods and compared with log-rank tests.

**Results:**

This analysis included 1,406 individuals. Across all periods, most patients were men aged 50–69 and presented with Lauren’s intestinal subtype. The frequency of stage IV disease significantly decreased between 2000–2004 and 2015–2017 (43.6% to 32.8%, *p* < 0.001). In contrast, we observed a rise in stage II (9.4% to 24.8%, *p* < 0.001) in the same comparison. We noticed an increased utilization of a combined approach involving chemotherapy and surgery (12% in 2000–2004 and 36.3% in 2015–2017, *p* < 0.001). The predicted 5-year OS of patients with GA in 2000–2004 was 27.8%, which increased to 53.9% in 2015–2017 (*p* < 0.001).

**Conclusion:**

Our retrospective cohort showed an upward trend in survival rates during the period. We observed that 5-year OS almost doubled among men and women during 2000–2017.

**Mini Abstract:**

The present retrospective cohort showed an upward trend in survival rates during the period from 2000 to 2017, in which the OS almost doubled among men and women.

## Introduction

Gastric cancer (GC) is the fifth most common cancer in Brazil [1] and worldwide [2], ranking fourth in global cancer-related mortality [3]. There has been a decrease in the global incidence and mortality of GC over the past decades [4, 5]. Nevertheless, the incidence has increased among young adults below the age of 50 [6].

Gastric adenocarcinoma (GA) constitutes approximately 90% of the total cases of GC [7]. Significant progress has been achieved in the management of GA over the past two decades [8, 9]. Curative treatment often involves a multimodal approach comprising R0 resection, D2 lymphadenectomy, radiotherapy and chemotherapy [10]. For advanced-stage patients, palliative systemic therapy is the standard of care, though it remains associated with an unfavourable prognosis [11].

Improvements range from adequate clinical staging with modern imaging techniques [12, 13] and diagnostic laparoscopy [14], to perioperative chemotherapy (PCT) for locally advanced disease [15, 16], and minimally invasive surgery [17]. Furthermore, systemic chemotherapy [18] and targeted agents [19] have increased survival in the advanced setting.

There is still a paucity of real-life data on GA survival analysis from underrepresented countries. Herein, we aim to describe the survival trends in patients with GA, regardless of their clinical staging, treated at a large cancer center in Brazil during a period of 17 years (2000–2017).

## Methods

This is a retrospective hospital-based cohort of patients diagnosed with GA between 2000 and 2017 who were treated at A.C. Camargo Cancer Center. All patients were identified from the Hospital Cancer Registry Database using ICD C16; data was extracted on 10 August 2022.

Patients were considered eligible if they were 18 years old or above and had histological confirmation of GA independent of clinical staging. The date of diagnosis was determined as the date of histopathological analysis. We excluded individuals with esophageal squamous-cell carcinoma, gastrointestinal stromal tumours, gastric lymphomas and other rare histologies. The primary objectives were to describe the patient population demographics, clinicopathological characteristics, treatment modalities and survival trends during four distinguished periods of diagnosis (2000–2004; 2005–2009; 2010–2014 and 2015–2017).

Age group (<50, 50–69, 70+) and gender were analysed. We also collected the following information about the disease: histopathological type (according to Lauren’s classification), and clinical staging, according to the American Joint Committee on Cancer (AJCC) 7th Edition. Treatment modalities were grouped as follows: surgery alone, radiotherapy alone, chemotherapy alone or any combination of these approaches.

Descriptive statistics were used for demographics, clinicopathological characteristics and treatment modalities. *χ*2 tests were used to compare categorical variables between two specified periods (2000–2004 and 2015–2017).

Overall survival (OS) was calculated from diagnosis to date of death by any cause or last follow-up. Survival curves were stratified by four separate periods (2000–2004; 2005–2009; 2010–2014 and 2015–2017) and by sex. 5-year OS was estimated using Kaplan-Meier curves, and comparisons were performed with log-rank tests. All tests were considered statistically significant with a two-sided *p*-value of < 0.05.

Statistical analyses and 5-year survival probability curves were performed with IBM SPSS Statistics (version 23). The study protocol was approved by the institutional ethics committee (n. 2462/17) on 12 May 2017.

## Results

The current analysis comprises a retrospective cohort of 1,406 patients (552 female and 854 male individuals). Demographics, clinicopathological characteristics and treatment modalities across the four periods of diagnosis are summarised in Table 1. The number of new cases per year was 46.8 during 2000–2004, and increased to 103.6 during 2015–2017. Across all periods, most patients were men aged 50–69 years and predominantly presented with Lauren’s intestinal subtype.

There were significant differences between staging groups during the study. Specifically, between 2000 and 2004, stage IV varied from 43.6% to 32.8% in 2015–2017 (*p* < 0.001). The most substantial variation in nonmetastatic cases occurred in stage II, comprising 9.4% of cases from 2000 to 2004 and rising to 24.8% in 2015–2017 (*p* < 0.001).

Concerning treatment modalities, we observed an increased utilisation of a combined approach involving chemotherapy and surgery throughout the investigation. Specifically, from 2000 to 2004, only 12% of patients received a multimodal treatment regimen consisting of chemotherapy and surgery, whereas, between 2015 and 2017, this proportion increased to 36.3% (*p* < 0.001). Also, patients receiving surgery + radiotherapy + chemotherapy varied from 12.4% of patients in 2000–2004 to 4.5% in 2015–2017 (*p* < 0.001).

**Figure 1.**
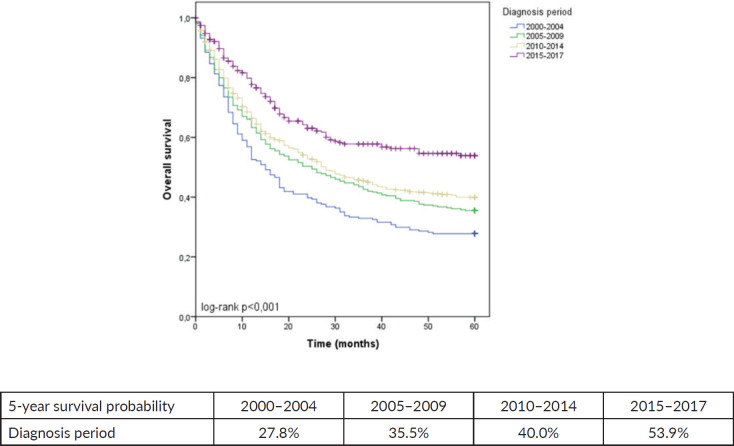
5-year OS for GA stratified by diagnosis period.

**Figure 2.**
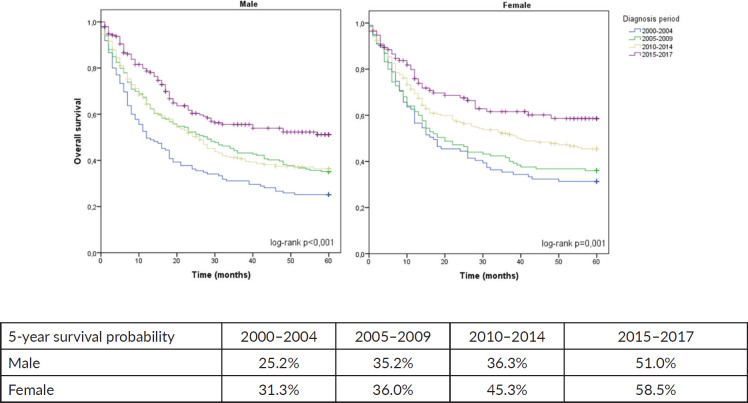
5-year OS for GA stratified by sex and diagnosis period.

When the entire cohort was analysed, the predicted 5-year OS of patients with GA between 2000 and 2004 was 27.8%, which increased to 53.9% in the period from 2015 to 2017 (*p* < 0.001), as shown in Figure 1. Among females, 5-year OS increased from 31.3% between 2000 and 2004 to 58.5% between 2015 and 2017 (*p* = 0.001). Among males, 5-year OS increased from 25.2% to 51.0% in the same comparison (*p* < 0.001), as shown in Figure 2.

## Discussion

This study represents a pioneering investigation of GA utilising a hospital-based cancer registry data in the Latin American context. Our findings describe the OS trends over 17 years in a tertiary cancer center. This milestone research contributes to understanding GA outcomes in our region, shedding light on critical aspects of patient prognosis and healthcare management.

The findings from our study reveal a consistent and notable increase in OS during the analysed period. This improvement may be attributed to several contributing factors, including a shift to earlier diagnoses, enhanced staging accuracy, more effective chemotherapy protocols, and improvements in surgical interventions. These factors collectively may elucidate the observed positive trends in survival outcomes.

Diagnosis of GA at earlier stages is related to higher OS [20, 21]. Our analysis revealed a significant trend towards more initial tumours, especially within AJCC 7th ed. clinical stage II. Although current guidelines do not recommend screening in the ‘average-risk’ population of Western countries [22], the more widespread availability of upper endoscopy [23] was an important factor leading to this transition of earlier staging GA cases.

Patients diagnosed with GA need a comprehensive staging evaluation to inform treatment decisions and enhance prognostication. In patients with locally advanced disease, the systematic use of staging laparoscopy detects occult peritoneal dissemination in 31% of patients [24]. A previous publication from our group highlighted that 57.7% of all GA patients underwent staging laparoscopy at our center [25].

International guidelines in Western countries recommend, as a preference, performing PCT as a multimodal approach [10, 26]. The pivotal Magic trial [16], published in 2006, compared perioperative Epirrubicin, Cisplatin and 5-Fluorouracil (ECF) versus surgery alone in 503 locally advanced gastroesophageal cancer patients in the United Kingdom. PCT significantly improved OS (HR, 0.75; 95%CI, 0.60–0.93; *p* = 0.009) and progression-free survival (HR, 0.66; 95CI, 0.53–0.81; *p* < 0.001).

Another randomised trial from France [27] compared doublet PCT with Cisplatin and 5-Fluorouracil, versus surgery in 224 locally advanced gastroesophageal cancer patients. PCT also demonstrated improved OS (5-year rate 38% versus 24%; HR, 0.69; 95%CI, 0.50–0.95; *p* = 0.02). At our institution, patients with locally advanced disease (mainly >cT3 and cN+) have been systematically referred to PCT, since 2006. Our previously published data revealed that 65.5% of nonmetastatic patients underwent PCT [28].

More recently, perioperative 5-Fluorouracil, Oxaliplatin and Docetaxel (FLOT) were compared to ECF in a phase 2/3, randomised trial [29, 30] in Germany. Patients with locally advanced gastroesophageal cancer (>cT2 or cN+) were included. Perioperative FLOT was significantly superior to ECF in OS (HR, 0.77; 95%CI, 0.63–0.94 *p* = 0.012). The period of this publication aside, FLOT has become our standard protocol since 2017.

Surgery remains the cornerstone of curative-intended treatment. The main goals of surgery are achieving an R0 resection [31] and performing a D2 lymphadenectomy [32]. In recent years, minimally invasive techniques, such as laparoscopic and robotic procedures, have become available. Surgical teams’ expertise and the perioperative care protocols are also highly relevant. Previously published data from patients treated at our cancer center demonstrated a low surgical mortality rate of 4.4% [25]. Also, the frequency of adjuvant chemoradiation, evaluated in the Intergroup US 0116 Trial [33], decreased during the period. It is known that this adjuvant strategy is less common in the present day due to standard D2 lymphadenectomy, which was performed in less than 10% of pivotal trials. Previously published from our cancer center demonstrated that D2-lymphadenectomy was performed in 90.8% of the patients [34].

To the best of our knowledge, this analysis represents the largest real-life GA database in our country. There might be some imprecision in collected information because of the extensive period covered in our research and changes made in the AJCC staging system over that period. It is also important to highlight that most of our patients were treated in the context of a private hospital. Hence, our results cannot be extrapolated to patients in the public health system. Moreover, interesting findings related to differences in OS, such as better survival in females, have not been fully clarified thus far and warrant further investigation.

## Conclusion

Our hospital-based data shows an upward trend in survival rates across patients with GA. We observed that 5-year OS almost doubled among men and women during the analysed period. Recent improvements in staging methods and treatment have increased cure rates. Nevertheless, further investigation is essential to determine significant differences in OS between males and females.

## Conflicts of interest

The authors declare no conflicts of interest.

## Funding

None.

## Figures and Tables

**Table 1.** Demographics, clinicopathological characteristics and treatment modalities during four separate periods of diagnosis.

Variable	2000–2004 *N* = 234	2005–2009 *N* = 324	2010–2014 *N* = 537	2015–2017 *N* = 311	*p-value
Sex					
Male	135 (57.7)	199 (61.4)	323 (60.1)	197 (63.3)	0.185
Female	99 (42.3)	125 (38.6)	214 (39.9)	114 (36.7)	
Age					
<50	52 (22.2)	61 (18.8)	100 (18.6)	45 (14.5)	0.064
50–69	109 (46.6)	141 (43.5)	274 (51.0)	160 (51.4)	
70+	73 (31.2)	122 (37.7)	163 (30.4)	106 (34.1)	
Histologic type					
Intestinal	98 (41.9)	133 (41.0)	210 (39.1)	118 (37.9)	0.093
Diffuse	75 (32.1)	98 (30.2)	202 (37.6)	102 (32.8)	
Undetermined	60 (25.6)	88 (27.2)	114 (21.2)	80 (25.7)	
Other	1 (0.4)	5 (1.5)	11 (2.0)	11 (3.5)	
Clinical staging					
I	33 (14.1)	63 (19.4)	85 (15.8)	55 (17.7)	<0.001
II	22 (9.4)	34 (10.5)	106 (19.7)	77 (24.8)	
III	45 (19.2)	64 (19.8)	93 (17.3)	53 (17.0)	
IV	102 (43.6)	125 (38.6)	218 (40.6)	102 (32.8)	
No data	32 (13.7)	38 (11.7)	35 (6.5)	24 (7.7)	
Treatment					
Surgery	96 (41.0)	91 (28.1)	105 (19.6)	56 (18.0)	<0.001
Radiotherapy	14 (6.0)	8 (2.5)	3 (0.6)	3 (1.0)	
Chemotherapy	31 (13.2)	58 (17.9)	133 (24.8)	73 (23.5)	
Surgery + Radiotherapy	5 (2.1)	2 (0.6)	-	2 (0.6)	
Surgery + Chemotherapy	28 (12.0)	69 (21.3)	190 (35.4)	113 (36.3)	
Radiotherapy + Chemotherapy	5 (2.1)	15 (4.6)	28 (5.2)	26 (8.4)	
Surgery + Radiotherapy + Chemotherapy	29 (12.4)	54 (16.7)	40 (7.4)	14 (4.5)	
Other treatment combination	-	-	-	10 (3.2)	
No treatment performed	26 (11.1)	27 (8.3)	38 (7.1)	14 (4.5)	

* *X^2^* test between 2000 and 2004 and 2015-2017. *p* < 0.05
